# Phthalates Not in Plastic Food Packaging

**DOI:** 10.1289/ehp.114-a89

**Published:** 2006-02

**Authors:** Patricia A. Enneking

**Affiliations:** American Plastics Council Arlington, Virginia E-mail: patty_enneking@plastics.org

I am writing with regard to a misleading photograph and caption in the article “Children’s Centers Study Kids and Chemicals” (Phillips 2005) published in the October 2005 issue of *EHP.* The article includes the following caption (p. A 665):

**Mothers, babies, and chemicals.** Researchers are studying whether variations in the enzymes that metabolize the phthalates found in some plastic bottles correlate with later birth and growth outcomes.

Above the caption is a photograph of a mother-to-be holding a plastic water bottle. Contrary to both the photograph and caption, phthalates are not used in plastic beverage bottles, nor are they used in plastic food wrap, food containers, or any other type of plastic food packaging sold in the United States.

The term “phthalates,” short for “orthophthalates,” refers to a class of additives that are used in some plastic products, specifically products made with a particular type of plastic—polyvinyl chloride (PVC or vinyl)—to make the material soft and flexible. Vinyl shower curtains, cable, wire, and flooring are examples of flexible PVC products that can contain phthalates.

Plastic beverage bottles sold in the United States are made from a type of plastic known as polyethylene terephthalate (PET). Although polyethylene terephthalate (the plastic) and phthalate (the additive) may have similar names, the substances are chemically dissimilar. PET is not considered an orthophthalate, nor does PET require the use of phthalates or other softening additives.

Another article in the same issue, “Are EDCs Blurring Issues of Gender?” (Hood 2005), echoes this misperception concerning phthalates and plastic food packaging. The article, which discusses phthalates, contains a photograph of plastic beverage bottles (p. A675) and, in the last two paragraphs (p. A677), makes reference to both plastic wrap and Saran Wrap. As a point of clarification, phthalates are not used in plastic food wraps sold in the United States categorically, and SC Johnson’s website specifically states that “… phthalates are not used in any Saran or Ziploc product” (SC Johnson 2006). The article (Hood (2005) also discusses bisphenol A, a substance used to make the plastic in some reusable water bottles, but not the convenience-size beverage packaging shown in the photograph.

The American Plastics Council respectfully requests that *EHP* address the misinformation that appeared in these articles and which is available on the *EHP* website.
